# Arginase: Mechanisms and Clinical Application in Hematologic Malignancy

**DOI:** 10.3389/fonc.2022.905893

**Published:** 2022-06-23

**Authors:** Zefan Du, Tianwen Li, Junbin Huang, Yun Chen, Chun Chen

**Affiliations:** ^1^ Department of Pediatrics, the Seventh Affiliated Hospital of Sun Yat-Sen University, Shenzhen, China; ^2^ Edmond H. Fischer Translational Medical Research Laboratory, Scientific Research Center, The Seventh Affiliated Hospital, Sun Yat-sen University, Shenzhen, China

**Keywords:** arginase, hematologic malignancy, mechanisms, clinical application, therapy

## Abstract

Compared to normal tissues and cells, the metabolic patterns of tumor illnesses are more complex, and there are hallmarks of metabolic reprogramming in energy metabolism, lipid metabolism, and amino acid metabolism. When tumor cells are in a state of fast growth, they are susceptible to food shortage, resulting in growth suppression. Using this metabolic sensitivity of tumor cells to construct amino acid consumption therapy does not harm the function of normal cells, which is the focus of metabolic therapy research at the moment. As a non-essential amino acid, arginine is involved in numerous crucial biological processes, including the signaling system, cell proliferation, and material metabolism. Rapidly dividing tumor cells are more likely to be deficient in arginine; hence, utilizing arginase to consume arginine can suppress tumor growth. Due to the absence of arginine succinate synthase, arginine succinate lyase, and ornithine carbamoyl transferase in some blood tumors, arginases may be employed to treat blood tumors. By investigating the mechanism of arginase treatment and the mechanism of drug resistance in greater depth, arginase treatment becomes more successful in hematological cancers and a new anti-cancer agent in clinical practice.

## Introduction

As a heterogeneous disease, the metabolic patterns of tumors differ significantly from normal tissues (1). Metabolic reprogramming of tumor cells refers to the metabolic changes caused by gene mutations and structural changes in tumor cells, such as enhanced glycolysis exemplified by the ‘Warburg effect,’ increased glucose uptake and consumption, increased amino acid uptake and catabolism such as glutamine, and increased lipid and protein synthesis. These metabolic alterations will contribute to the malignant proliferation of tumor cells and the adaptation to an unfavorable living environment (2–6). The distinction between tumor cell and normal cell metabolic pathways encourages the development of antitumor therapies based on metabolic pathways (7). Metabolic reprogramming allows tumor cells to thrive in the microenvironment of nutritional deficiency and hypoxia, but there are also metabolic vulnerabilities. Compared to normal cells, tumor cells are dependent on exogenous nutrients, especially amino acids (8, 9). The metabolic reliance of tumor cells on exogenous amino acids has become the theoretical foundation for the creation of amino acid deprivation therapy (9).

**Figure 1:**
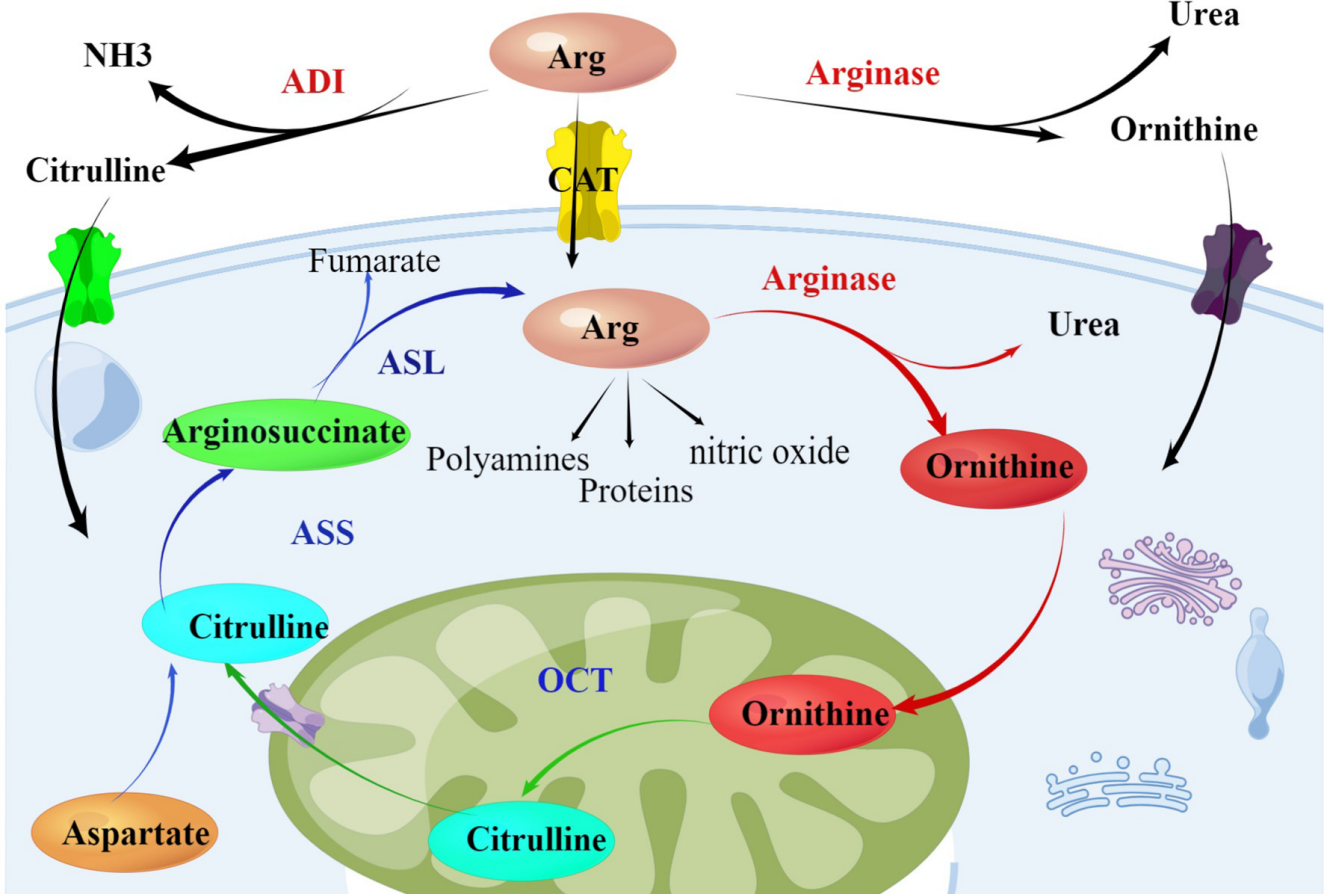
Arginine metabolism within the urea cycle: Arginase catalyzes the hydrolysis of arginine into ornithine and OCT catalyzes the synthesis of citrulline from ornithine. Aspartate and citrulline are condensed by the enzymatic activity of ASS and the resulting intermediate arginosuccinate is then split by ASL, producing again arginine. Tumor cells lacking the enzyme ASS rely on exogenous supply of arginine. The endogenous sources of arginine can be used as a potential therapeutic intervention targets in treating cancer. Arg, Arginine; ADI, arginine deiminase; ASS, argininosuccinate synthetase; ASL, argininosuccinate lyase; OCT, ornithine carbamoyltransferase.

Arginine is a multifunctional amino acid, involved in many important biological functions in the body, including cell proliferation, signal transduction, muscle growth, nerve transmission, etc., involved in protein synthesis, nucleotide synthesis, urea cycle and other important metabolic pathways, and as a prerequisite for the synthesis of many molecular substances such as citrulline, nitric oxide, glutamic acid, proline biosynthesis (10, 11). Arginine is also classified as a semi-essential amino acid in human body. Under normal physiological conditions, there are endogenous and exogenous sources of arginine, that is, the production of arginine from the starting through ornithine cycle and the uptake of extracellular arginine by cells through membrane protein transporters (12). The key enzymes for arginine synthesis and metabolism mainly include arginine succinate synthase (ASS), arginine succinate lyase (ASL) and ornithine carbamoyl transferase (OCT) (**Figure 1**). The low expression of these key enzymes makes it impossible for many malignant tumors to synthesize arginine endogenously and become arginine deficient tumor cells, including many hematological malignancies (13). Arginine auxotrophic tumors can be treated by arginine-depleting enzymes without interfering with normal cell physiology, thus becoming a potential antitumor strategy (14).

As an enzyme that depletes arginine, arginase has become a crucial component of arginine depletion treatment. It has been shown to have an anti-tumor impact on hematological malignancies, mostly by triggering autophagy, apoptosis, oxidative stress, and even interfering with the progression of the cell cycle to induce tumor cell death. As a result, this introduction focuses on the current state of arginase application in hematological cancers, associated research mechanisms, and its potential as a treatment for hematological tumors.

## Arginine Nutritional Deficiencies and Deprivation Therapy

The fundamental aspect of tumor cells is metabolic reprogramming, which is to adapt to their rapid multiplication by modifying their metabolic processes, as well as the survival milieu of hypoxia and nutritional shortage (15). During the process of metabolic reprogramming, it is also susceptible to dietary deficiencies, particularly for the growth of essential nutrients like amino acids (16). Currently, amino acid deprivation therapy for amino acid nutritional deficit has become a promising anti-tumor therapy and has yielded positive outcomes in numerous tumor treatments. The concept of amino acid deprivation was developed in the therapy of tumors, and L-asparaginase was launched more than 50 years ago for the treatment of juvenile leukemia. L-asparaginase is currently one of the most important medications for the combination chemotherapy of juvenile acute lymphoblastic leukemia, which enhances the remission rate of this disease (17, 18). As a result of the efficacy of asparaginase in the therapy of leukemia, there are numerous metabolic enzymes that investigate amino acid nutritional deficits, including enzymes that target arginine metabolism, glutamine, and cysteine.

As a semi-essential amino acid, arginine participates in numerous cellular biological processes, transforms into other amino acids (such as glutamine, proline, etc.), and creates proteins, urea, and agmatine (19).On a biological level, the primary sources of arginine in plasma are dietary digestion and absorption and other protein modification, whereas intestinal-kidney axis arginine synthesis contributes for just 5-15 percent. In the *de novo* synthesis of arginine, ornithine and carbamoyl phosphate are catalyzed by OCT to produce citrulline, which is subsequently catalyzed by ASS1 and ASL to produce L-arginine. The rate-limiting enzymes of endogenous arginine synthesis are ASS1 and ASL, which are typically expressed in normal cells and unaffected by arginine consumption (19, 20).

Due to the low or non-expression of essential enzymes in arginine endogenous synthesis, especially ASS1, ASL, and OCT, in numerous cancers, tumor cells cannot ordinarily generate endogenous arginine and are highly dependent on exogenous arginine; these tumors are referred to as arginine-auxotrophic. Studies reveal that more than 70 percent of cancers, including melanoma, hepatocellular carcinoma, renal cell carcinoma, glioblastoma, and prostate cancer, as well as a variety of hematological tumors, have arginine shortage (21–23). Cai Y et al. investigated the expression level of the ASS1 gene in various malignancies using the UALCAN database and determined that the expression level of ASS1 in acute myeloid leukemia (AML) was the lowest of all cancers, thereby making AML a typical arginine auxotrophic tumor (24). In addition to Hodgkin’s lymphoma and non-Hodgkin’s lymphoma, low expression of ASS1 was observed in additional solid hematological malignancies (22).

Current studies on the genetic mechanism of low expression of ASS1 gene have confirmed that methylation of CPG regions within the ASS1 gene promoter may be a common epigenetic mechanism of ASS1 gene silencing expression (23), or may include other mechanisms such as inhibition of ASS1 promoter by hypoxia inducible factor HIF-1α. Through the study of glioblastoma cases, Syed N et al. found that the CpG island of ASS1 with methylation was up-regulated in the sensitivity to arginine deprivation therapy, further verifying that the expression of ASS1 was affected by the methylation of gene promoter (25). In the study of melanoma cells by Tsai WB et al., it was found that the regulation of ASS1 expression was caused by the interaction between the negative regulator HIF-1α and the positive transcription regulator c-Myc and Sp4. HIF-1α combined with the E-box site at the promoter of ASS1 gene could induce the silence of ASS1 expression and cause arginine consumption sensitivity (26). These related studies further induced epigenetic mechanisms by exploring the expression of ASS1 and ASL genes, thus providing ideas for the development of new treatments.

Arginine depletion therapy for arginine-auxotrophic tumors by arginine depletion enzyme (ADE) paves the way for a new anti-tumor treatment. In scientific research of arginine metabolism, the primary degrading enzymes are arginine decarboxylase (ADC), arginine deiminase (ADI), and arginase. ADI is mostly generated from prokaryotes, and ornithine cannot be synthesized *via* the ADI route as a source of energy for their growth. Mycoplasma-derived ADI is the first antitumor medication to be documented. Even though it has some therapeutic effects, it is easily detected and inactivated by the immune system because of its immunogenicity. So ADI must be bioengineered into PEG-ADI in clinical settings to minimize its immunogenicity (27). ADC, an additional arginine-depriving enzyme, is mostly expressed in mammals. Due to its cytotoxicity to healthy normal cells, it is not employed as a cancer treatment at this time. In contrast to these two enzymes, the arginases currently under investigation are derived from humans and lack immunogenicity and high toxicity. Currently, it has been demonstrated that they have potential therapeutic benefits on a variety of solid and hematological malignancies (11, 28).

## Arginase and its Application in Hematological Malignancy

Arginase is a manganese-dependent metal ion hydrolase that catalyzes arginine metabolism to generate non-toxic products ornithine and urea. Human tissues express two distinct kinds of arginase, arginase I and arginase II, which are encoded by two distinct genes on separate human chromosomes (29). Human arginase I is mostly found in the cytoplasm of liver cells and is primarily responsible for the consumption of excess ammonia in human tissues *via* the urea cycle, whereas human arginase II is a mitochondrial enzyme with high expression e.g.in kidney tissue. Arginase II is primarily involved in the creation of nitric oxide and polyamines, as opposed to the arginine urea cycle (30, 31).

In the initial investigation, the arginase was purified from animal liver; however, due to the high dose need of purified arginase treatment, further research and application are limited (32). Human arginase was PEGylated to produce recombinant PEGylated arginase (rhArg-peg), and the catalytic activity of arginase was enhanced by substituting cobalt ions for manganese ions. Its half-life and activity time were lengthened, and its immunogenicity was diminished (33, 34). RhArg has therapeutic potential for malignancies lacking OCT or ASS expression. According to studies, arginine deiminase is ineffective in tumors expressing ASS, mostly because arginine can be synthesized from citrulline *via* ASS and ASL (33, 35).

Although arginase therapy in hematological malignancies has not yet reached the approved clinical stage, many studies have confirmed the potential therapeutic activity of arginase in hematological malignancies. Francis Mussai et al. confirmed that pegylated recombinant human arginase (BCT-100) led to rapid consumption of arginine inside and outside cells, thereby inducing cell cycle arrest and cell death. They further revealed that it is because of defective expression of ASS and OCT in AML cells (36). In addition to the therapeutic effect in AML cells, BCT-100 treatment can block the expression of cyclin D3 in cells, thereby inducing apoptosis of T- acute lymphoblastic leukemia (T-ALL) cells *in vitro* and *in vivo*, reflecting the potential therapeutic effect of BCT-100 on T-cell malignancies by consuming arginine (28). In addition, Carmela De Santo et al. showed that BCT-100 combined with dexamethasone had obvious cytotoxicity on acute lymphoblastic leukemia (ALL) cells. Although ALL cells up-regulated the expression of arginine transporter CAT-1 and endogenous ASS or OCT, it still could not prevent the cytotoxicity of BCT-100 (37). The combination of BCT-100 and cytarabine has been effective in the treatment of AML and ALL *in vivo* and *in vitro*, but drug resistance easily develops, so that further research is needed. In hematological solid tumors, Zeng X et al. demonstrated that recombinant human arginase can inhibit the growth of non-Hodgkin’s lymphoma (NHL) cells by arginine deprivation, which may play an anti-tumor role by cell cycle arrest and caspase-dependent apoptosis (38).

## Effect of Arginase Treatment on Hematological Malignancy

Using recombinant arginase to treat nutritionally deficient hematological cancers is expected to trigger cell death, which may be mediated by autophagy, cell cycle arrest, or oxidative stress. After treatment with recombinant human arginase, studies have demonstrated that apoptosis results in cell death in ALL, but AML exhibits caspase-independent and non-apoptotic cell death (39). In the research of the influence of BCT-100 on the cell cycle and proliferation, Francis Mussai et al. discovered that the G0/G1 phase of AML cells treated with BCT-100 remained unchanged. Compared to untreated cells, the expression of cyclin A was substantially elevated, whereas the expression of cyclins B and E was lowered, resulting in cell cycle arrest, necrosis, and death (36).

The increased generation of ROS is the cause of tumor cell death produced by BCT-100. Studies demonstrate that BCT-100 therapy further induces ROS generation in bladder cancer cells, blocking phosphorylation of AKT and mTOR and triggering autophagy and death of tumor cells (39). S. Xu et al. also discovered that rhArg slows tumor cell proliferation *via* autophagy, cell cycle arrest, and oxidative stress in the research on hepatocellular carcinoma and breast cancer (40).

## Study on the Resistance Mechanism of Arginase

In arginase-treated cells, the expression of ASS1 is restored and up-regulated, allowing arginine-deprived tumor cells to recover endogenous arginine synthesis. c-MYC is a positive regulator of ASS1 expression, and its activation is the primary cause of ASS1 reexpression (41). Arginine deprivation enhances ROS generation in the tumor microenvironment, which in turn activates Gas6/Axl to initiate the PI3K/Akt/GSK3B pathway, resulting in c-Myc oncogene expression upregulation (42). By replacing the negative regulator HIF-1a of the ASS1 gene promoter and competitively occupying the E box of the promoter, up-regulated c-Myc restores and up-regulates the expression of the ASS1 gene, thereby restoring the endogenous synthesis of arginine (43).

Dietary restriction can trigger autophagy in cells. Through autophagy, cells can recruit damaged proteins and defective organelles into autophagosomes for digestion and breakdown misfolded proteins or organelles by binding to lysosomes, thereby supplying the energy and materials necessary for cell survival. Also present in arginase treatment is autophagy. Short-term arginine deprivation therapy decreases ATP and NO levels, induces endoplasmic reticulum stress by activating the mTOR pathway, promotes cell autophagy, and nourishes cells with critical nutrients. In the absence of arginine for an extended period of time, cancer cells can activate the Bax-mediated apoptotic pathway or re-express the ASS gene to produce resistance to arginases (44, 45). Zeng X et al. confirmed that recombinant human arginase activated autophagy in lymphoma cells, human promyelocytic leukemia cells, and human T-ALL cells, which served a protective function (38). In practice, the combination of autophagy inhibitors (such as 3-methyladenine and chloroquine) can augment the anti-tumor activity of arginase and induce apoptosis in tumor cells (46).

In addition to the aforementioned potential resistance mechanisms, arginase therapy is associated with the activation of several metabolic pathways. During the development of arginase resistance, proteomics reveals that the cellular metabolism shifts from glutamine dependence to glucose dependence (47, 48). Several investigations have demonstrated that once the arginase consumes arginine, cell growth becomes dependent on aspartic acid. Aspartic acid is the precursor of pyrimidine nucleotides and can be used as the substrate for arginine synthesis by ASS. Therefore, arginase therapy should be combined with different metabolic inhibition treatments to achieve better therapeutic effects, which requires additional research (48).

## Conclusion

As a semi-essential amino acid in the human body, arginine participates in a variety of vital biological processes. By arginine-degrading enzymes, arginine depletion therapy can limit the growth of nutritionally deficient tumors. Hematological cancers, including AML, ALL and various lymphomas, have been shown to respond favorably to this treatment. Through recombinant engineering, arginase has been transformed into pegylated recombinant human arginase, which has a longer half-life, and less adverse effects. Currently, the application of arginase to hematological cancers has entered the clinical research phase, and additional investigation is required to confirm the therapeutic benefit. Drug resistance mechanisms of arginase therapy is still in its exploratory phase, and more study is required to clarify it. To develop more effective treatment options for hematological cancers based on arginase, more in-depth discussion and study is required.

## Author Contributions

Contributions: Conception and design: YC, CC; Administrative support: None; Provision of study materials or patients: None; Collection and assembly of data: None; Data analysis and interpretation: None; Manuscript writing: All authors; Final approval of manuscript: All authors.

## Acknowledgments

We thank Sanming Project of Medicine in Shenzhen (No. SZSM202011004), and Shenzhen Healthcare Research Project (Grant No. SZLY2018001) for supporting the manuscript preparation and publication.

## Conflict of Interest

The authors declare that the research was conducted in the absence of any commercial or financial relationships that could be construed as a potential conflict of interest.

## Publisher’s Note

All claims expressed in this article are solely those of the authors and do not necessarily represent those of their affiliated organizations, or those of the publisher, the editors and the reviewers. Any product that may be evaluated in this article, or claim that may be made by its manufacturer, is not guaranteed or endorsed by the publisher.
